# Oynophagia in patients after dental extraction: surface electromyography study

**DOI:** 10.1186/1746-160X-2-34

**Published:** 2006-10-17

**Authors:** Michael Vaiman, Oded Nahlieli, Eli Eliav

**Affiliations:** 1 Chief Surgeon, Department of Otolaryngology, Assaf Harofeh Medical Center, affiliated to the Sackler Faculty of Medicine, Tel Aviv University, Tel Aviv, Israel; 2 Professor and Chairman, Dept. of Oral and Maxillofacial Surgery, Barzilai Medical Center, Ashkelon, Israel, and Hebrew University-Hadassah School of Dental Medicine, Jerusalem, Israel; 3 Associated Professor, UMDNJ – New Jersey Dental School, Newark, NJ, USA

## Abstract

**Objectives:**

Surface electromyographic (sEMG) studies were performed on 40 adult patients following extraction of lower third and second molars to research the approach and limitations of sEMG evaluation of their odynophagia complaints.

**Methods:**

Parameters evaluated during swallowing and drinking include the timing, number of swallows per 100 cc of water, and range (amplitude) of EMG activity of m. masseter, infrahyoid and submental-submandibular group. The above mentioned variables (mean + standard deviation) were measured for the group of dental patients (n = 40) and control group of healthy adults (n = 40).

**Results:**

The duration of swallows and drinking in all tests showed increase in dental patients' group, in which this tendency is statistically significant. There was no statistically significant difference between male and female adults' duration and amplitude of muscle activity during continuous drinking in both groups (p = 0.05). The mean of electric activity (in μV) of m. masseter was significantly lower in the dental patients' group in comparison with control group. The electric activity of submental-submandimular and infrahyoid muscle groups was the same in both groups.

**Conclusion:**

Surface EMG of swallowing is a simple and reliable noninvasive method for evaluation of odynophagia/dysphagia complaints following dental extraction with low level of discomfort of the examination. The surface EMG studies prove that dysphagia following dental extraction and molar surgery has oral origin, does not affect pharingeal segment and submental-submandibular muscle group. This type of dysphagia has clear EMG signs: increased duration of single swallow, longer drinking time, low range of electric activity of m. masseter, normal range of activity of submental-submandibular muscle group, and the "dry swalow" aftereffect. The data can be used for evaluation of complaints and symptoms, as well as for comparison purposes in pre- and postoperative stages and in EMG monitoring during treatment of post-surgical oral cavity discomfort and dysphagia.

## Background

For decades the investigation of dysphagia has been concentrated on evaluation of single and separate swallows of normal subjects and neurological or otolaryngological patients [1-5]. The same tendency is true also for research activities with EMG evaluation of deglutition [6-9]. Dysphagia, however, is a common symptom in dentistry as well. It can appear following dental extraction [10], bimaxillary osteotomy [11], odontogenic infection [12], and other dental problems [13]. The connection between dentistry and dysphagia becomes more prominent in the elderly [14].

The single swallow and continuous drinking tests are important not only in evaluation of dysphagia, but also in evaluation of odynophagia (for example, water drinking test after tonsillectomy) and in differential diagnosis in cases of dysphagia of unknown origin. In case of dental surgery, odynophagia and dysphagia are usually related [15,16]. Dysphagia, or difficulty with swallowing, is defined as any defect in the intake or transport of endogenous secretions and nutriments necessary for the maintenance of life. Odynophagia is a painful swallowing. While dysphagia can be with or without pain, odynophagia it its turn can produce secondary dysphagia as patients trying to reduce pain change their normal swallowing patterns. Numerous studies were performed in this field; yet, clinicians still indicate the need for a simple noninvasive test for assessment of postsurgical dysphagia/odynophagia complaints.

The needle electrode EMG technique provides important information to physicians, but, being invasive, in case of face and neck locations it has its limitations and can be used more for scientific purposes. In contrast, the sEMG technique is suitable for routine clinical diagnostic use. We tried to show that surface EMG evaluation of dysphagia following dental surgery is clinically useful, can provide valuable data for diagnosis and monitoring of dysphagia/odynophagia and simple to perform at dental departments.

## Materials and methods

### Subjects

The patients were studied across a 4-month period. The study was approved by the Medical Center Ethics Committee in regard to Helsinky Protocol. The group of dental patients who undergone unilateral lower second or third-molar surgery included 40 adults, 22 women and 18 men, ranging in age from 18 to 71 years (mean = 31.9 years). This group was randomly chosen by a sealed envelope method from 143 dental patients with similar diagnosis. The control group included 40 healthy adult volunteers (F 24, M16, age mean = 29.7 years), usually relatives of the patients. Before the study all subjects completed a questionnaire regarding their general health and their medical history. Subjects had no history of dysphagia or odynophagia prior dental surgery (for patients), and no history of medical problems or medications that might affect swallowing and drinking (for control group) (inclusion criterion). All subjects had normal oral anatomical structures and complete dentition with an exception of operated site (patients) (inclusion criterion). None of patients and healthy volunteers had a history or symptoms of abnormality or disease of tempomandibular joint or any respiratory diseases, which might affect breathing (exclusion criteria). All subjects were assessed by the dental surgeons prior to their participation to the study to rule out possible oral abnormalities other than operated site (exclusion criterion). All the males were well shaved. At the time of the test the subjects reported they were not thirsty.

### Electromyographic techniques

Three muscle locations were examined in the study: (1) m. masseter, (2) the submental-submandibular muscle group that includes anterior belly of digastric, mylohyoid, and geniohyoid, and (3) infrahyoid muscle group, all covered by platisma. These muscles were selected because they are superficial and they are thought to be involved in the oral and pharyngeal phases of the swallow.

All EMG recordings were made using standard surface electrodes (AE-131). The equipment used for the EMG recordings was a NeuroDyne Neuromuscular Sys/3 four channel computer based EMG unit with NeuroDyne Medical software (NeuroDyne Medical, Mass, USA), and AE-204 Active sensors attached to AE-131 electrodes. The unit has wide bandpass filter, bandwidth (RMS) 25–450 Hz and 60 Hz notch filter. The system uses the Active Electrode, a compact sensor assembly that includes a miniaturized instrument preamplifier. Locating the amplifier at the electrode site allows artifacts to be cancelled and the signal boosted before being transferred down the electrode cable. The integration period of the sEMG signal at the hardware level is 25 milliseconds. This has very little effect on the shape of the signal. For the software, the underlying sampling speed is 100 Hz (100 samples per second). This high speed sampling is then typically averaged based on the Sampling Rate that is selected. Each EMG record was full-wave rectified and low-passed filtered. The computer program indicates mean, standard deviation, minimum, maximum, range of muscle activity during each trial, and its duration. Muscle activity (EMG) is quantified in microvolts.

**Figure 1:**
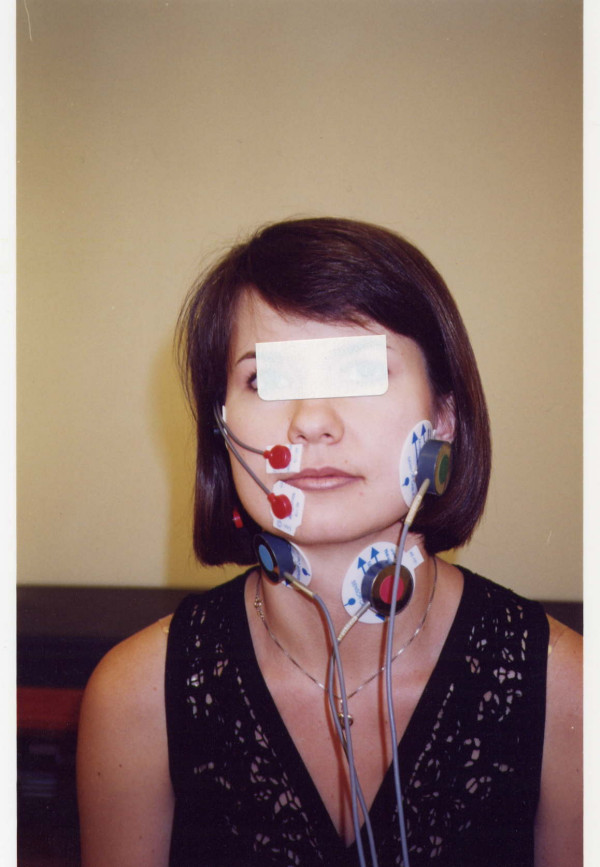
A subject with masseter, submental-submandibular and infrahyoid muscles locations of EMG electrodes. The picture also shows the orbiculars oris location of electrodes. This location, while being less informative, can be used if some additional information on oral function is required.

The interelectrode distance was 10 mm. Specific electrode positions were as follows (Fig. 1): (1) Two bipolar stick-on surface electrodes were placed parallel to the masseter muscle fibers on the side opposite to the operated side of the face. (2) Two surface electrodes were attached to the skin beneath the chin on the right or left side of midline (beneath the operation site) to record submental myoelectrical activity over the platisma. (3) Two electrodes were placed on the left side of the thyroid cartilage to record from the infrahyoid (laryngeal strap) muscles. The exact electrode positions for each muscle are known since the 19th century [17,18], and in addition were clarified following anatomical correlates [19]. Each pair of electrodes had a third electrode as ground. In case of AE-131, the common (ground) electrode is designed close to the differential (active) electrodes. Electrical impedance at sites of electrode contact was reduced, as target areas were lightly scrubbed with alcohol gauze pads, followed by application of an electrode gel.

### Procedures

Two tests were examined: voluntary single water swallows as normal from an open cup and continuous drinking of 100 cc of tap water from an open cup. In the group of dental patients the tests were examined 24 hours and 72 hours after surgery. After the two pairs of sEMG electrodes were attached, subjects remained completely relaxed for a minute in order to establish the sEMG visual pattern for resting potential of facial and neck muscles. Subjects were permitted to move chin slightly up while swallowing if needed, as we traced no changes of the graphic and numerical baseline associated with this movement. (This movement involves mm. rectus capitis posterior minor and minor, as well as some other posterior neck muscles and does not affect signals from above mentioned electrode locations.) The single swallow test was performed after a mean volume of the normal swallow bolus was calculated for group of healthy volunteers (control group).

After electrode placement, each participant performed two tasks:

1. Three trials of swallowing mean volume of tap water from an open cup. The volume, calculated before the trials, was 15.5 cc per single swallow. Instruction given: "Swallow this water in one gulp".

2. After that subjects performed a trial of continuous drinking of 100 cc of tap water. Instruction given: "Drink this water as normal".

A total of six swallows and two drinking period were obtained per participant. Totally 480 swallows and 160 drinking periods were evaluated during this study. The graphic records were then evaluated. Ten subjects were re-tested a day later to detect intertrial difference for the duration variable. These subjects were chosen randomly (sealed envelope method) from the dental patients' group. Interjudge reliability was assessed by comparing scores obtained for each swallowing trial for each of the two tasks. Two judges blinded to group assignment were involved and the test observer agreement was good (Kappa coefficient 0.81).

In order to evaluate how do obtained sEMG graphic patterns reflect the sequence of physiological events during deglutition, for five volunteers the sEMG testing was performed with simultaneous fluoroscopy with barium contrast.

We examined single swallowing and continuous drinking of 100 cc of tap water from an open cup (duration, mean electric activity of muscles, type of sEMG graphic record, and number of swallows). The data were analysed off-line by computer. All graphic recordings were initially inspected by eye. The data were statistically evaluated with SPSS software, Standard version 10.0.5 (SPSS, Chicago, IL, 1999). A χ^2 ^criterion using 95% confident interval was used to compare categorical variables, and *t*-test to compare continuous variables. The level of significance for all analyses was set at p < 0.05. The α level was set at 0.05.

## Results

**Figure 2:**
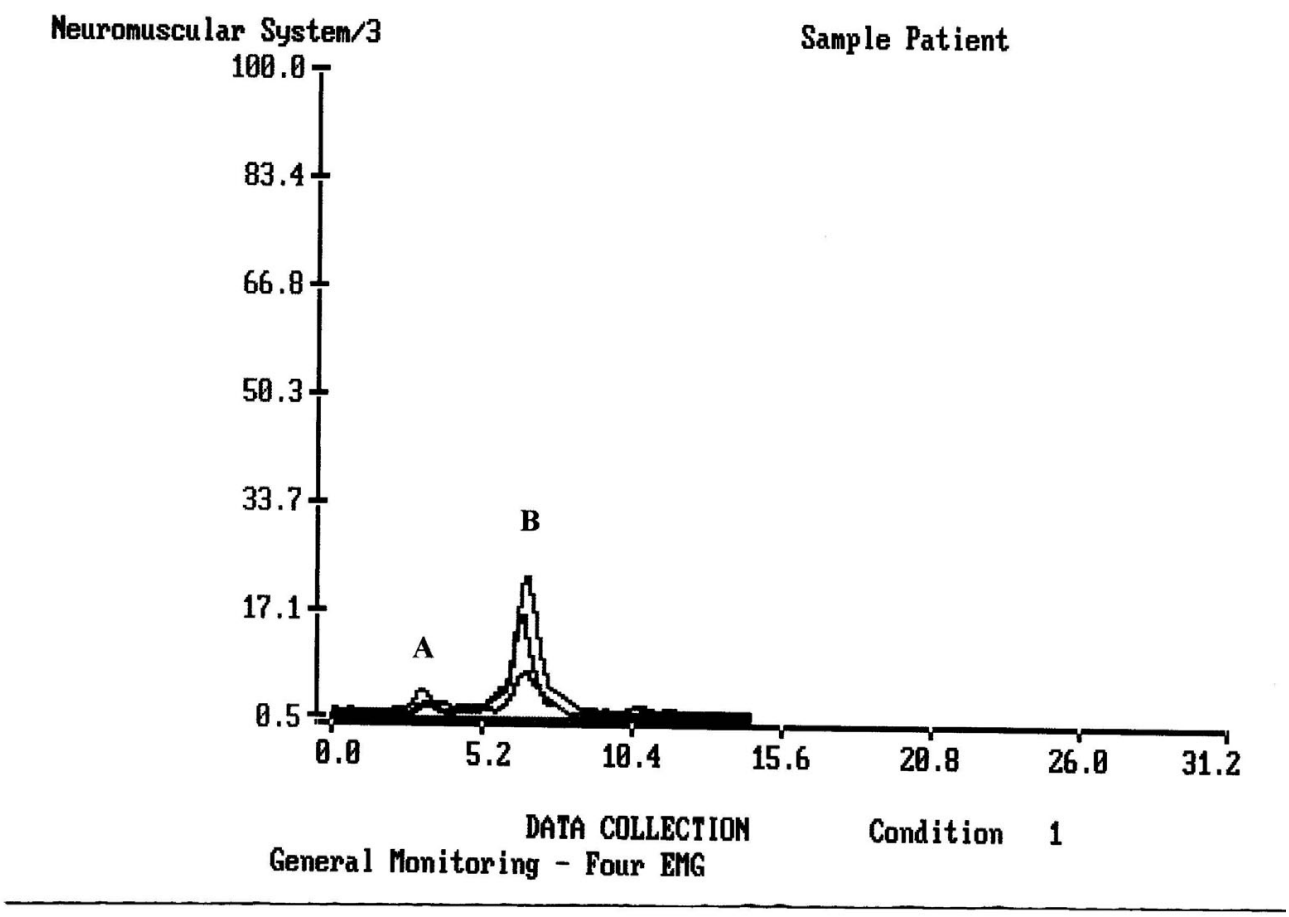
A normal swallow of a healthy person. A – labial seal, B – a swallow. Upper peak – submental-submandibular location, middle peak – masseter location, lower peak – infrahyoid location.

**Figure 3:**
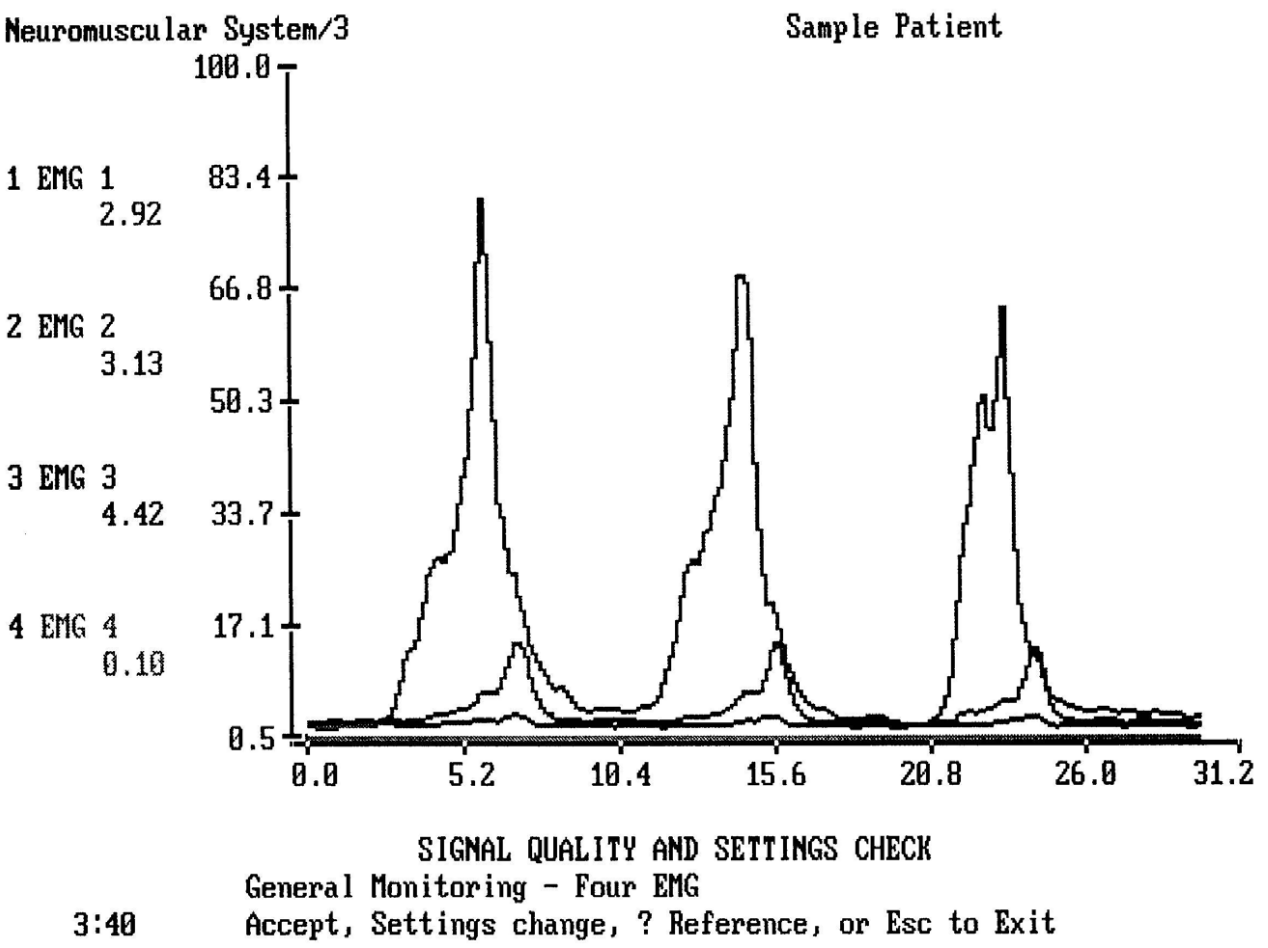
A typical test of three single normal swallows of a person, 24 hours after dental surgery. The complete electric activity of muscles during swallow is longer (here 5.21 sec) that normal swallow of a healthy person. The submental and infrahyoid peaks are normal (upper and middle lines), the masseter line (lower line) shows the lack of peaks. The Masseter almost does not work.

The results indicated that the range and mean of sEMG activity as well as its duration varied greatly among subjects (Figs 2 and 3).

The duration of the reflex phase of muscle activity during single swallows showed prolonged swallow in the group of dental patients. This tendency was statistically significant if compared to healthy volunteers (p < 0.01, condition × duration). In this test, for healthy adults (control group) the mean duration of muscle activity in the recorded reflex phase of normal swallow was 3.44 sec. For the group of dental patients this duration was 4.59 sec (p < 0.05). These times represent the duration of sEMG activity which lasts longer then actual time required to pass a bolus from the oral cavity to the esophagus.

The data for mean and range of EMG activity of masseter, infrahyoid and submental-submandimular muscles during single swallow test are shown in the Table 1. In this test, the mean activity at submental location was 30–50% higher than the activity at masseter location for control group, and 85–95% higher for the group of dental patients, i.e. m. masseter was almost inactive after dental surgery. The infrahyoid location showed no significant tendency. In the group of dental patients, the electric activity of masseter (range, in μV) recorded 72 hours after dental surgery was 35% higher than the activity recorded 24 hours after the surgery. The activity of submental-submandibular and infrahyoid muscles remained the same.

**Table 1:** Range of electric activity of m. masseter, submental-submandibular and infrahyoid muscles in a single swallow test, in μV (mean ± standard deviation)

	Control group	Dental patients	
		24 h postop	72 h postop

TESTS:			
**Masseter: Normal swallow**	17.124 ± 7.548	11.875 ± 4.183	15.045 ± 5.762
**Submental: Normal swallow**	36.750 ± 10.032	25.754 ± 9.025	27.650 ± 8.333
**Infrahyoid: Normal swallow**	16.183 ± 6.954	10.875 ± 3.045	15.873 ± 3.775

In the act of continuous drinking, the one gulp water intake can be measured by dividing 100 cc into the number of swallows the individual performed and this can be recorded on an EMG system. The mean volume of liquid per swallow during continuous drinking was 14.7 cc for the control group, and only 9.4 cc for the group of operated patients. We monitored a clear tendency for a decrease of the mean volume of liquid per swallow for the operated dental patients (p < 0.01).

For the group of dental patients, we monitored a tendency for an increase of the duration of muscle activity. This tendency was significant if compared with the control group (p < 0.005). Specifically, the mean duration of drinking of 100 cc of water was 10.8 sec for the control group, and 18.7 sec for the dental patients group. The number of swallows for drinking 100 cc of water also increased for the dental patients group (p = 0.01) and this was accompanied by a simultaneous decrease of amount of water per swallow (p = 0.01) (Table 1).

**Figure 4:**
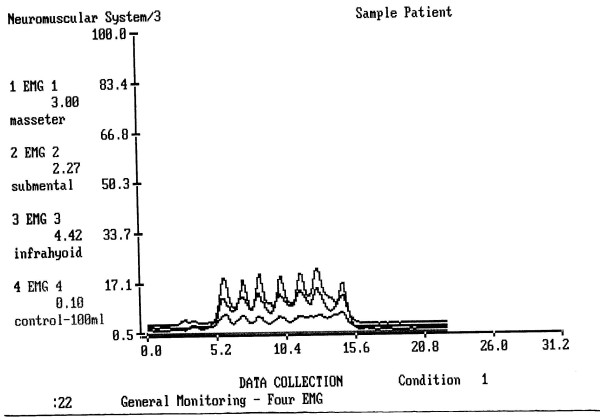
An example of normal drinking of 100 cc of water. It took this subject 11.04 sec to drink 100 cc of water in 7 swallows. Upper line = the submental-submandibular electrode location, middle line = the masseter electrode location, lower line = the infrahyoid location. All these muscles are almost completely relaxed before and after drinking.

**Figure 5:**
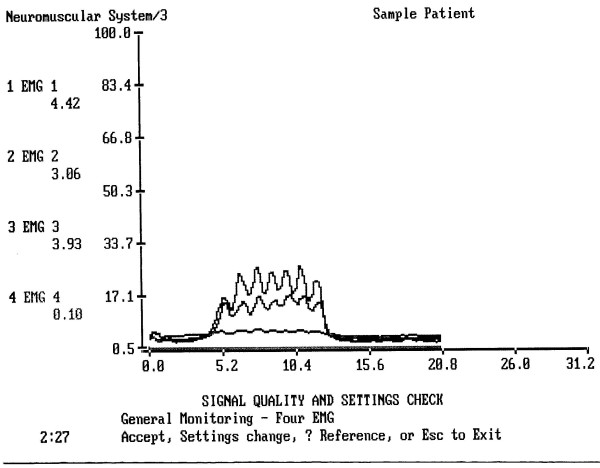
An example of drinking of 100 cc of water 24 hours after a dental surgery. Upper line = the submental-submandibular electrode location, middle line = the infrahyoid electrode location, lower line = the masseter location. The masseter line shows no peaks and no elevation during drinking.

The mean electrical activity of m. masseter, infrahyoid and submental-submandibular muscle group (covered by the m. platysma) during the test of continuous drinking of 100 cc of water is shown in Table 2. In healthy painless drinking, the submental-submandibular location amplitude of electric activity was insignificantly higher than the amplitude of the m. masseter. In the group of dental patients the amplitude of m. masseter was so low, that this difference became significant (p < 0.001). The infrahyoid location was less informative for comparison. (Figs. 4, 5).

**Table 2:** Continuous drinking of 100 ml water – duration of muscle activity excluding the initial oral stage (mean ± SD)

	Control group	Dental patients
Total duration (s)	10.2 ± 2.78	17.9 ± 5.32
Number of swallows	6.9 ± 1.8	10.7 ± 2.90
Duration of one swallow (*s*)	1.51 ± 0.4	1.75 ± 0.6
ml/swallow	14.5 ± 3.55	10.0 ± 2.85
ml/swallow in %	100%	69%

A significant number of healthy subjects (7, 17,5%, p < 0.05) performed a dry swallow after normal drinking. It occurred when all the water had already been swallowed. The volunteers described this act as being "compelling" and, in essence, were unaware of having done it. Among these 7 volunteers, this additional dry swallow was observed in 71.43% of the drinking episodes (i.e., 7 volunteers × 2 drinking episodes each = 14 periods, of which 10 periods were accompanied by a final dry swallow and 4 were not). In the group of dental patients we found the dry swallow effect in 20 subjects (50%, p < 0.005), significantly more frequent than in a control group (p < 0.01), and occured in 92.5% of drinking episodes.

There was no statistically significant gender related difference for the duration and mean muscle activity during both tests for both groups (p = 0.12).

## Discussion

In our departments, we use the sEMG method for quick evaluation of patients'complaints on dysphagia/odynophagia and for localization of the etiologic agent of the suffering. For example, in case if dysphagia and/or odynophagia were caused by odontogenic infection in the submandibular space, the traces from submandibular-submental location rather than from masseter location will show some abnormalities. It also helps us in differential diagnosis. For example, if dysphagia was caused by salivary gland dysfunction [20], the traces from masseter will be higher in amplitude in contrast with dental extraction, when as we saw, the amplitude of muscle activity of masseter is very low. The above described method can be used for differential diagnosis of dysphagia cases caused by implants, local anaesthetics, etc. In addition to this, we use EMG for monitoring of healing after oral surgery and tracing postsurgical complications.

Graphically a typical single water swallow of a healthy individual was observed at the rectified and low-passed filtered sEMG as a normal wave with upward deflections and sharp apex when recorded from both masseter and submental-submandibular locations. The beginning of water swallow is usually seen as a mild elevation of the line and represents the final oral stage of a swallow which occurs when the tongue is moved so as to squeeze the liquid volume against the hard palate. Submental muscles and masseter support the tongue-induced pressure. At this stage the automatic reflexive gesture of swallowing is triggered. The same record taken from a patient after dental surgery showed normal waves at submental-submandibular and infrahyoid locations and much lower wave, or almost no activity at all, at the masseter location.

This phenomenon clearly shows that the dysphagia in dental patients is of oral origin and does not affect pharyngeal stage of a swallow. The patients initially have postoperative odynophagia. Trying to reduce the pain, they change their chewing and swallowing patterns. Thus, reducing odynophagia to a tolerated minimum, they get dysphagia instead demonstrating longer duration of swallow, smaller bolus selection, and sEMG changes. In fact, this type of dysphagia is secondary to odynophagia. A patient tries to spare the operated site, does not clench teeth and, therefore, does not involve masseter in the acts of swallowing and drinking. We did not trace significant left-right differences of masseter activity in operated persons during the water swallowing or drinking tests when chewing is not involved. That is why we usually place MS electrode to the side opposite to the operated side (stick-on electrodes are to be pressed against the skin). It is done in order not to cause additional painful sensations in the patients.

The amount of water was set at 100 cc, approximately a half of a standard glass. The lesser volume, for example, 50 cc, can be swallowed in two gulps and no reliable data can be obtained. If a volunteer drinks a full glass of 200 cc of water, swallowing/ventilation interactions are significant and can affect the validity of obtained data.

Since the discoveries of F. Magendie, the act of single swallowing is ordinary described as consisting of three stages: oral, pharyngeal, and oesophageal [21,22]. Sometimes the oral stage is divided into oral initial and oral final stages [23]. Therefore, dysphagia can be of oral, oropharyngeal, or pharyngoesophageal.

The results of the study showed that the group of patients after lower molar surgery demonstrated significant difference between them and the group of volunteers in respect of m. masseter involvement in the act of swallowing. In addition to the clear tendency for bolus volume decrease in continuous drinking swallows, these group demonstrate prolonged duration of single swallows and longer drinking time for 100 cc of water. Drinking the same amount of water, the patients after dental surgery make almost twice as more swallows in comparison to healthy volunteers.

In respect to dry swallow aftereffect, we feel that it occurs because of some triggering irritation sometimes remaining in the throat and being enough to initiate additional reflexive action. Some studies [24] described a feedback during swallowing between pharyngoesophageal segment and the oral cavity. It can, perhaps, explain the high frequency of this phenomenon among patients after dental surgery.

Surface EMG signals of swallowing may be influenced by many variables like neck size, body mass and skin condition. These variables, however, mainly affect resting potential data, less affecting the duration of drinking. At the same time, if a patient has an unshaved bristle, his recorded resting potential (baseline) will be high. In evaluation of continuous drinking range (amplitude of a signal) is not important because all the involved muscles do not relax completely between swallows, and the real range of swallow cannot be estimated in contrast with a single swallow test.

Also during sEMG testing, a certain amount of impedance noise arises directly from the resistance of the electrodes' connection to the skin. It makes skin resistance a significant factor when working with the low level EMG signals typical to small muscles involved in swallowing. Wiping the skin with isopropyl alcohol in water solution has proven to be the best form of preparation for most situations. The alcohol removes the dead skin and surface oils, and the water moistens the skin and provides improved ion flow. The sEMG sensors we used are designed so that using of electrode gel is not obligatory.

## Conclusion

Surface EMG of swallowing is a simple and reliable method for evaluation of single swallowing and continuous drinking with low level of discomfort of the examination. There is no statistically significant difference between male and female adults' duration of muscle activity during single swallowing and continuous drinking in healthy individuals and examined dental patients. The duration of the reflex phase of swallows shows significant increase in patients who presented symptoms of dysphagia after lower molar surgery. The timing of events and amplitude data can be used for diagnostic and differential diagnostic purposes, objective evaluation of complaints, as well as for comparison purposes in pre- and postoperative stages, monitoring of postsurgical healing, tracing postsurgical complications and in EMG monitoring during dental treatment.

**Table 3:** Mean of electric activity (in μV) of the masseter, submental-submandibular and infrahyoid muscle groups covered by the platysma during a continuous drinking test of 100 cc of water.

	Control group	Dental patients
**M. masseter**		
Raw mean*	6.122	7.359
SD	2.310	2.514
Real mean**	3.627	4.864
**The submental-submandibular muscle group**		
Raw mean	10.238	12.658
SD	2.174	3.186
Real mean	7.430	9.850
**The infrahyoid group**		
Raw mean	6.012	5.884
SD	1.696	1.935
Real mean	3.204	3.076

*Raw mean = computer-calculated mean

**Real mean = raw mean minus the mean resting potential of an actual muscle covered by skin (2.808 μV = for the m. submental and m. infrahyoid, 2.495 μV = for the m. masseter, and 4.542 = for the m. orbicularis oris).
